# Pregnancy-Recognized Obstructive Sleep Apnea and Maternal–Fetal Outcomes: A Narrative Review of Gestational Rhinitis, Upper-Airway Mechanisms, and Treatment Evidence

**DOI:** 10.3390/healthcare14213598

**Published:** 2026-10-23

**Authors:** César Alas-Pineda, Gustavo Ferrer, Dennis J. Pavón-Varela, Kristhel Gaitán-Zambrano, Carlos Coto-Tejeda, Jhacely Medina-Mejía, Mark L. Cannon

**Affiliations:** 1 Department of Research and Development, Moxie Health Group, Hallandale Beach, FL 33009, USA; dennisjpavon@gmail.com (D.J.P.-V.); kmelissag13@gmail.com (K.G.-Z.); carloseduardocoto@gmail.com (C.C.-T.); jhacely2000@outlook.es (J.M.-M.); 2 Department of Pulmonary and Critical Care Medicine, Aventura Hospital and Medical Center, Aventura, FL 33180, USA; gferrer@pulmonary-institute.com; 3 Division of Dentistry, Department of Otolaryngology, Feinberg School of Medicine, Northwestern University, Chicago, IL 60611, USA; markcannon@northwestern.edu

**Keywords:** sleep apnea, pregnancy complications, rhinitis, pre-eclampsia, pregnancy outcome

## Abstract

**Highlights:**

**What are the main findings?**

• Pregnancy-recognized obstructive sleep apnea (POSA) is associated with hypertensive disorders and preterm birth in observational studies; an association with impaired fetal growth is possible, but residual confounding remains important.
• A 2024 meta-analysis, including randomized and observational studies, found the use of continuous positive airway pressure to be associated with a reduction in gestational hypertension and preeclampsia; the preeclampsia estimate was not robust in subgroup and sensitivity analyses, and several small randomized trials found no statistically significant differences in their main outcomes; certainty remains limited by adherence, study size, and inclusion of observational evidence.

**What are the implications of the main findings?**

• In selected high-risk pregnant patients, risk-based evaluation and objective testing may be considered in accordance with current consensus guidance.
• Rhinitis therapies used in pregnancy have supportive gestational safety data, but their efficacy for gestational rhinitis has not been demonstrated, and they should not be considered OSA treatment.

**Abstract:**

**Background/Objectives**: Pregnancy-recognized obstructive sleep apnea (POSA) is an operational construct, not a validated biological phenotype, and remains underrecognized in prenatal care. This review synthesizes evidence on POSA-related outcomes, evaluates gestational rhinitis as a candidate contributor to upper-airway vulnerability, and distinguishes pregnancy safety from demonstrated efficacy for OSA-related endpoints. **Methods**: This structured narrative review, informed by SANRA criteria, used a structured PubMed search (7 March 2026) with targeted supplementary searches through 9 October 2026, across six predefined domains. **Results**: Pregnancy-recognized OSA is associated in observational studies with hypertensive disorders, preterm birth, impaired fetal growth (in small pregnancy-specific studies), and severe maternal morbidity; residual confounding by obesity precludes causal inference. In a 2024 meta-analysis of randomized and observational studies (*n* = 809), continuous positive airway pressure (CPAP) was associated with lower risks of gestational hypertension (RR, 0.65; 95% CI, 0.47–0.89) and preeclampsia (RR, 0.70; 95% CI, 0.50–0.98). Although secondary endpoints in individual trials suggested benefit, the pooled preeclampsia effect was fragile in sensitivity and subgroup analyses. Gestational rhinitis affects approximately one in five pregnant women and is a plausible but untested contributor to pregnancy-recognized OSA. Saline irrigation, budesonide (mainly inhaled-exposure data), and chlorpheniramine have gestational safety data, but the only randomized trial of an intranasal corticosteroid in gestational rhinitis was null, and we identified no study of OSA endpoints. **Conclusions**: Given the largely observational evidence, objective testing and individualized treatment may be considered in high-risk patients, pending adequately powered trials. CPAP is the standard treatment for objectively confirmed OSA.

## 1. Introduction

Obstructive sleep apnea (OSA), first recognized during pregnancy—pregnancy-recognized OSA (POSA)—is the specific focus of this review. Pregnancy-recognized OSA is an operational construct encompassing both genuinely de novo OSA precipitated by gestational physiological changes and pre-existing, previously undiagnosed OSA first coming to clinical attention during obstetric care; it does not imply a distinct biological phenotype, and Section 3 provides full definitional criteria. Despite documented associations between OSA and adverse outcomes in both mother and fetus, screening for sleep-disordered breathing (SDB) is not routinely implemented in prenatal care, and POSA continues to be systematically underrecognized [1].

The prevalence of SDB (encompassing habitual snoring, upper-airway resistance syndrome, and OSA) increases across gestation. Prospective polysomnographic studies estimate SDB prevalence at 17% to 45% in the third trimester. Nearly all available data defined SDB as an AHI or respiratory disturbance index ≥ 5 events/h using scoring rules; in one cohort, AHI ≥ 5 reported a prevalence of 66%, AHI ≥ 15 fell to 16%, and AHI ≥ 30 to 3% [2]. Separately, administrative data show that administratively coded OSA at delivery in the United States nearly doubled, from 8.5 to 16.6 per 10,000 hospitalizations, between 2016 and 2020 [3]. These data sources measure distinct constructs and are not directly comparable. The apparent increase in coded OSA may reflect greater clinical awareness and diagnostic testing or rising obesity, a diagnosis present in 69.7% of pregnant patients with coded OSA versus 11.4% of those without [3].

The clinical relevance of OSA in pregnancy derives from its maternal–fetal consequences. Pregnant women with coded or objectively confirmed OSA face higher rates of cesarean delivery, gestational hypertension, preeclampsia, and preterm birth in observational and administrative database studies [1,3]. Prospective cohort data suggest that OSA may be associated with impaired fetal growth, but available pregnancy-specific studies are small, and the association was attenuated and no longer statistically significant after adjustment for body mass index [4]. In a secondary analysis of Nulliparous Pregnancy Outcomes Study: Monitoring Mothers-to-Be (NuMoM2b) Sleep-Disordered Breathing, including 2106 nulliparous individuals, Delgado et al. found that new-onset mid-pregnancy AHI-defined SDB was associated with a higher incidence of adverse neonatal composite outcomes in multivariable analysis (adjusted RR 1.43, 95% CI 1.05–1.94) [5]. In contrast, the association between early-pregnancy AHI-defined SDB and adverse neonatal composite outcomes was no longer significant after full covariate adjustment. Residual confounding by obesity and metabolic comorbidities cannot be excluded, and the available observational study designs do not support causal inference. These associations, observed across study designs and populations despite heterogeneous OSA definitions, justify examining how pregnancy increases OSA susceptibility and which contributors warrant investigation.

Established physiological contributors to increased OSA susceptibility include gestational weight gain and fluid redistribution. In contrast, progesterone-mediated increases in ventilatory drive and upper-airway muscle activity may exert a countervailing protective effect [2,6]. These mechanisms are thought to contribute to the increase in SDB across gestation, particularly during the second and third trimesters, and raise the question of whether gestational rhinitis additionally contributes to upper-airway vulnerability [7].

Gestational rhinitis, originally defined as nasal congestion present during the last six or more weeks of pregnancy without signs of respiratory tract infection or a known allergic cause and disappearing completely within two weeks after delivery [8], has a reported cumulative incidence of approximately one in five pregnancies [9,10]. It may also contribute to upper-airway vulnerability in the context of pregnancy-recognized OSA, although this link has not been tested. We identified no study that measured pharyngeal collapsibility, nasal nitric oxide, or OSA severity in pregnant women with gestational rhinitis and concurrent SDB. Therefore, this review examines gestational rhinitis as a candidate rather than an established mechanism.

This review synthesizes evidence on the maternal–fetal consequences of pregnancy-recognized OSA, summarizes screening and diagnostic approaches (including diagnostic thresholds and guideline criteria for risk-based screening), evaluates gestational rhinitis as a hypothesis-generating contributor to upper-airway vulnerability, and appraises management options within a framework that distinguishes established gestational safety from confirmed efficacy for OSA-related endpoints. The main contribution of this review is integrating separate studies into a hypothesis-generating conceptual model, not a validated model of pregnancy-recognized OSA.

## 2. Materials and Methods

This structured narrative review was designed and reported with reference to the quality criteria of the Scale for the Assessment of Narrative Review Articles (SANRA) [11]. It is organized around four objectives: (i) synthesizing evidence on the maternal–fetal consequences of POSA; (ii) summarizing screening and diagnostic approaches, including guideline criteria; (iii) evaluating the biological plausibility of gestational rhinitis as a mechanistic contributor to upper-airway vulnerability; and (iv) appraising pharmacological management options within a framework that explicitly distinguishes gestational safety from efficacy for OSA-specific endpoints. Unlike prior reviews of POSA that addressed primarily obstetric outcomes, this review explicitly integrates the mechanistic role of gestational rhinitis and upper-airway physiology—including the biologically plausible but unconfirmed H1-mediated mucosal pathway—into the analysis of POSA, its susceptibility, and management. PubMed was used as the primary database because the review’s clinical and physiological domains are predominantly indexed in MEDLINE. To reduce potential single-database bias, particularly for drug-safety, prevalence, and CPAP evidence, we relied, where available, on systematic reviews, meta-analyses, and evidence-based guidelines, several of which searched additional databases [7,12,13,14]. The absence of Embase and the Cochrane Library searches is acknowledged in Section 11. The full Boolean strategy is provided in Appendix A.1. Although no review protocol was registered, an internal protocol defining the search domains, data synthesis, and inclusion criteria was defined before screening began and was not modified during study selection.

Six domain-specific search blocks were constructed using MeSH terms and text-word keywords in Boolean combination. The structured search was conducted on 7 March 2026, using the full strategy described in Appendix A.1. On 9 October 2026, we performed a targeted supplementary search, rather than a complete repetition of the predefined strategy, to verify statements of absence of evidence, prevalence, and diagnostic-accuracy estimates, and the trials included in a published meta-analysis [14]. Two independent reviewers (C.A.P. and K.G.Z.) screened the records and appraised the evidence, resolving disagreements through discussion. Methodological limitations were appraised qualitatively for each evidence domain, considering study design, sample size, exposure ascertainment, adjustment for confounding, and, for intervention studies, adherence and the analysis population. No formal risk-of-bias tool or structured certainty of evidence framework was applied, and no quantitative synthesis was performed. Narrative reviews and laboratory or physiological studies were used to provide background and to support mechanistic hypotheses, not to estimate clinical effects.

Inclusion criteria were: (i) human or animal studies reporting pathophysiology, prevalence, diagnostic accuracy, or outcomes of SDB during pregnancy, without year restriction; (ii) intervention studies of pharmacological or non-pharmacological SDB treatment in pregnant women, without year restriction; (iii) clinical practice guidelines and systematic or narrative reviews, with priority given to documents published from 2015 onward; and (iv) publications in English. Studies in non-pregnant adult populations were included when they provided foundational mechanistic data unavailable in the gestational context. Each such study is explicitly labeled by population in the text. Reference lists of included articles were hand-searched for additional sources.

Eighty-four records were initially retrieved; following deduplication (*n* = 13), 71 unique records were screened. After title, abstract, and full-text review, 33 studies and guidelines comprised the initial structured evidence set (Appendix A.2). During revision, the targeted supplementary searches described above identified six sources that the original search strategy did not retrieve [13,14,15,16,17,18]. One clinical review [9], one methodological reference [11], and one systematic review suggested during peer review [19] were added outside the structured PubMed search; the final manuscript cites 42 references. Appendix A.3 indicates where each SANRA item is addressed in this review.

When findings differed across study designs, we prioritized randomized trials, prospective cohorts with objective sleep testing, and adjusted estimates for BMI and metabolic comorbidities. For discordant results, we presented findings side by side rather than reconciling them into a single estimate, and we explicitly noted discrepancies.

## 3. Definitions

Sleep-disordered breathing (SDB) spans a spectrum from habitual snoring to upper-airway resistance syndrome to OSA. Obstructive sleep apnea (OSA) denotes recurrent complete or partial upper-airway obstruction during sleep and is commonly defined in clinical settings by an apnea-hypopnea index (AHI) ≥ 5 events per hour on polysomnography or home sleep apnea testing (HSAT), in conjunction with symptoms or relevant comorbidity; however, many pregnancy studies use AHI ≥ 5 events per hour alone as the case definition [2,20]. Pregnancy-recognized obstructive sleep apnea (POSA) is defined in this review as obstructive sleep apnea first recognized during pregnancy in a woman with no prior documented OSA diagnosis. This operational definition encompasses both genuinely de novo OSA precipitated by gestational physiological changes and pre-existing, previously undiagnosed OSA that first came to clinical attention during obstetric care. Pregnancy-recognized OSA reflects a diagnostic circumstance rather than a validated biological phenotype.

The standard diagnostic cut-off of AHI ≥ 5 events per hour was derived from studies in non-pregnant adults; some epidemiologic studies define OSA solely by an AHI threshold, without requiring concurrent symptoms or cardiovascular comorbidity, a distinction noted where relevant in the evidence summarized below. During pregnancy, the risks of hypertensive disorders and gestational diabetes increase across AHI categories [20]. The Society of Anesthesia and Sleep Medicine-Society for Obstetric Anesthesia and Perinatology (SASM-SOAP) 2023 consensus guideline defines OSA as an AHI ≥ 5 events/hour, but it does not establish a pregnancy-specific AHI threshold for treatment. Many recommendations for treating pregnant and postpartum patients are based on the standard of care for non-pregnant adults; the same treatments can be extrapolated (Class IIb recommendation; Level C-EO). CPAP is recommended to reduce OSA symptoms and AHI (Class IIa; Level C-LD/EO). SASM-SOAP recommends counseling pregnant patients with OSA that treatment targets symptoms, normalizes objective measures of OSA, and improves quality of life in general [7]. In this review, we use AHI ≥ 5 events/hour as the minimum diagnostic criterion for OSA, while acknowledging that adverse events have also been reported with milder SDB [2].

Table 1 summarizes the relationships among these four constructs—and the evidence types associated with each—and readers should consult it when interpreting the prevalence and outcome data presented in subsequent sections, as cross-construct confusion is a common source of misinterpretation in this literature.

## 4. Physiological and Hormonal Changes in Pregnancy Relevant to SDB

Pregnancy induces systemic immune and physiological changes, primarily driven by elevated progesterone and estrogen [2,7]. Gestational weight gain and fluid redistribution increase upper-airway vulnerability through worsening respiratory mechanics and pharyngeal collapsibility, and are associated with higher frequency and severity of apneic and hypopneic events across gestation; sleep position may further modulate event frequency in susceptible individuals [2]. OSA arises from recurrent upper-airway obstruction during sleep, producing intermittent hypoxia, sleep fragmentation, sympathetic activation, and endothelial dysfunction; these mechanisms may contribute to preeclampsia risk by dysregulating placental vascular function, as suggested by case–control biomarker data [21].

### Progesterone and Upper-Airway Patency

Progesterone is considered the principal gestational hormone with a potential protective effect on upper-airway patency. In non-pregnant women, awake genioglossus electromyographic activity was highest in the luteal phase of the menstrual cycle, correlated weakly with progesterone levels, and increased in postmenopausal women after two weeks of combined estrogen–progesterone replacement, whereas upper-airway resistance did not differ [6]. This hormonal influence may contribute to the lower OSA prevalence among premenopausal women than among men [6], and the rise in SDB across gestation occurs despite this potentially protective effect [2]. The net upper-airway consequence of pregnancy, therefore, reflects a balance between this protective progesterone effect and the multiple vulnerability factors introduced by gestational weight gain, fluid redistribution, and mucosal changes [2,6].

## 5. Gestational Rhinitis and Its Proposed Contribution to Obstructive Sleep Apnea: A Hypothesis-Generating Framework

Gestational rhinitis was originally defined as nasal congestion present during the last six or more weeks of pregnancy, without other signs of respiratory tract infection and with no known allergic cause, disappearing completely within two weeks after delivery [8]; in a prospective questionnaire-based study, it appeared at any time during gestation [10]. This definition distinguishes it from allergic rhinitis, which may predate pregnancy and is driven by IgE-mediated mechanisms, and from vasomotor rhinitis, in which neurogenic dysregulation predominates. The distinction has pharmacological relevance: the pathogenesis of gestational rhinitis is incompletely understood and probably multifactorial, with hormonally mediated vascular engorgement as the predominant proposed mechanism rather than classic IgE-mediated mast-cell degranulation [9]; H1 antagonists would therefore not be expected to address the full disorder or its dominant obstructive mechanism. When sneezing, rhinorrhea, or nasal pruritus accompany obstruction, concomitant allergic rhinitis or another rhinitis subtype should be considered; in that context, H1-directed therapy may be a rational, symptom-directed option for those symptoms [7,22].

Frequency estimates derive from Swedish cohorts: pregnancy rhinitis developed in 5 of 23 women followed with daily nasal recordings (22%) [8], and a questionnaire-based study of 599 women attending antenatal care reported a cumulative incidence of 22% [10]. In a retrospective series from a tertiary otolaryngology clinic, rhinitis of pregnancy accounted for 10 of 57 pregnant patients evaluated for rhinologic concerns (17.5%), a referral-based proportion rather than a population estimate [23]. Nasal obstruction is the dominant symptom; in a prospective study with daily recordings, subjective congestion scores were higher in early and late pregnancy than after delivery [9], and the late-pregnancy increase coincides temporally with the rise in SDB prevalence in the third trimester [2].

### 5.1. Nasal Mucosal Edema: Objective Physiological Data

**Figure 1:**
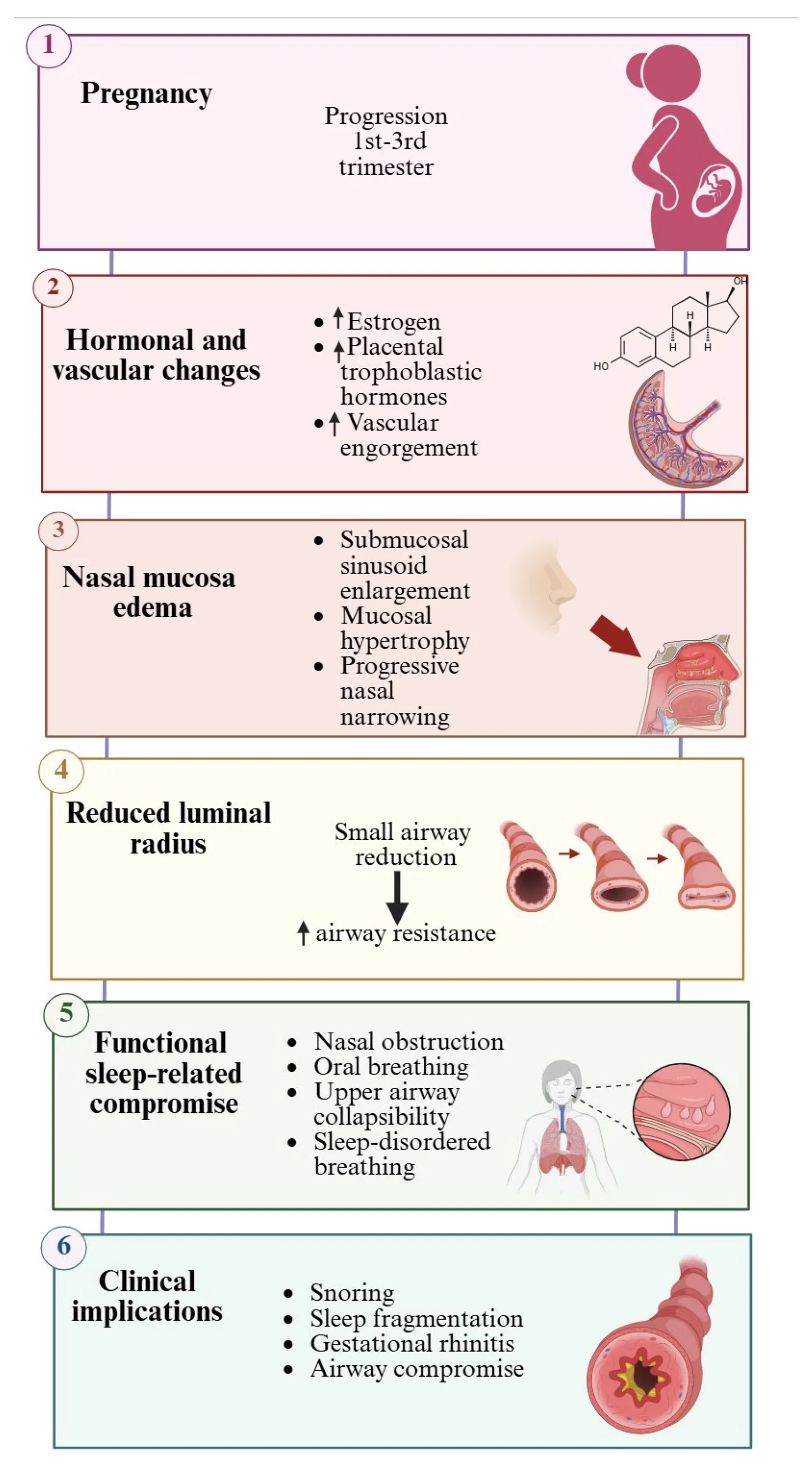
Proposed physiological and anatomical changes during pregnancy that may lead to nasal obstruction, and their potential clinical implications. The sequence is hypothesis-generating; objective longitudinal data on nasal patency in pregnancy are limited and inconsistent. Figure 1 created with BioRender under license.

Objective longitudinal data on nasal patency during pregnancy are scarce and inconsistent. In a prospective study of 23 pregnancies with daily nasal and oral peak expiratory flow recordings, objectively registered nasal blockage increased during pregnancy in eight women but declined in nine [8]. Vascular engorgement and mucosal changes attributed to gestational hormones have been proposed as the underlying mechanism [9]. Because small reductions in luminal radius produce disproportionate increases in resistance, pregnant women with rhinitis-grade obstruction may experience functionally significant nasal airway compromise during sleep without meeting criteria for structural nasal pathology such as polyp disease or septal deviation (see Figure 1) [24].

### 5.2. Proposed Mechanisms (Untested in Pregnancy)

The mechanistic relevance of gestational rhinitis to OSA rests on three pathways. Data are extrapolated from non-pregnant adults, in vitro studies, and physiological modeling, as none have been measured in pregnant women with concurrent rhinitis and SDB in the studies identified. They are presented to structure the research agenda in Section 10, not to inform clinical management.

Oral breathing and pharyngeal collapsibility. Nasal obstruction promotes oral breathing during sleep. In non-pregnant adults, upper-airway resistance during oral breathing is approximately 2.5-fold higher than during nasal breathing, and oral breathing is associated with greater pharyngeal collapsibility at the retropalatal and retroglossal levels [25]. Gestational rhinitis may produce variable nasal obstruction, possibly greater in the supine position and in late pregnancy, which could contribute to OSA pathogenesis by introducing night-to-night and position-dependent variability in pharyngeal loading; this has not been measured in pregnant women. Progesterone-associated augmentation of genioglossus activity [6] and of ventilatory drive [2] may provide a partial but likely incomplete countervailing effect. Consistent with, but not specific to, this proposed interaction, habitual snoring rises from about 7–11% in the first trimester to 16–25% in the third trimester across prospective cohort studies [2]; this temporal parallel does not establish a contribution of nasal obstruction.

Nasal nitric oxide. The paranasal sinuses produce NO in high concentrations, which is entrained into the inspiratory airstream and delivered to the lower airway, where it has been proposed to contribute to bronchodilation and ventilation-perfusion matching in non-pregnant subjects [25]. Oral breathing bypasses this delivery pathway, but we identified no studies of nasal NO in pregnant women with gestational rhinitis or SDB, representing a research gap.

**Figure 2:**
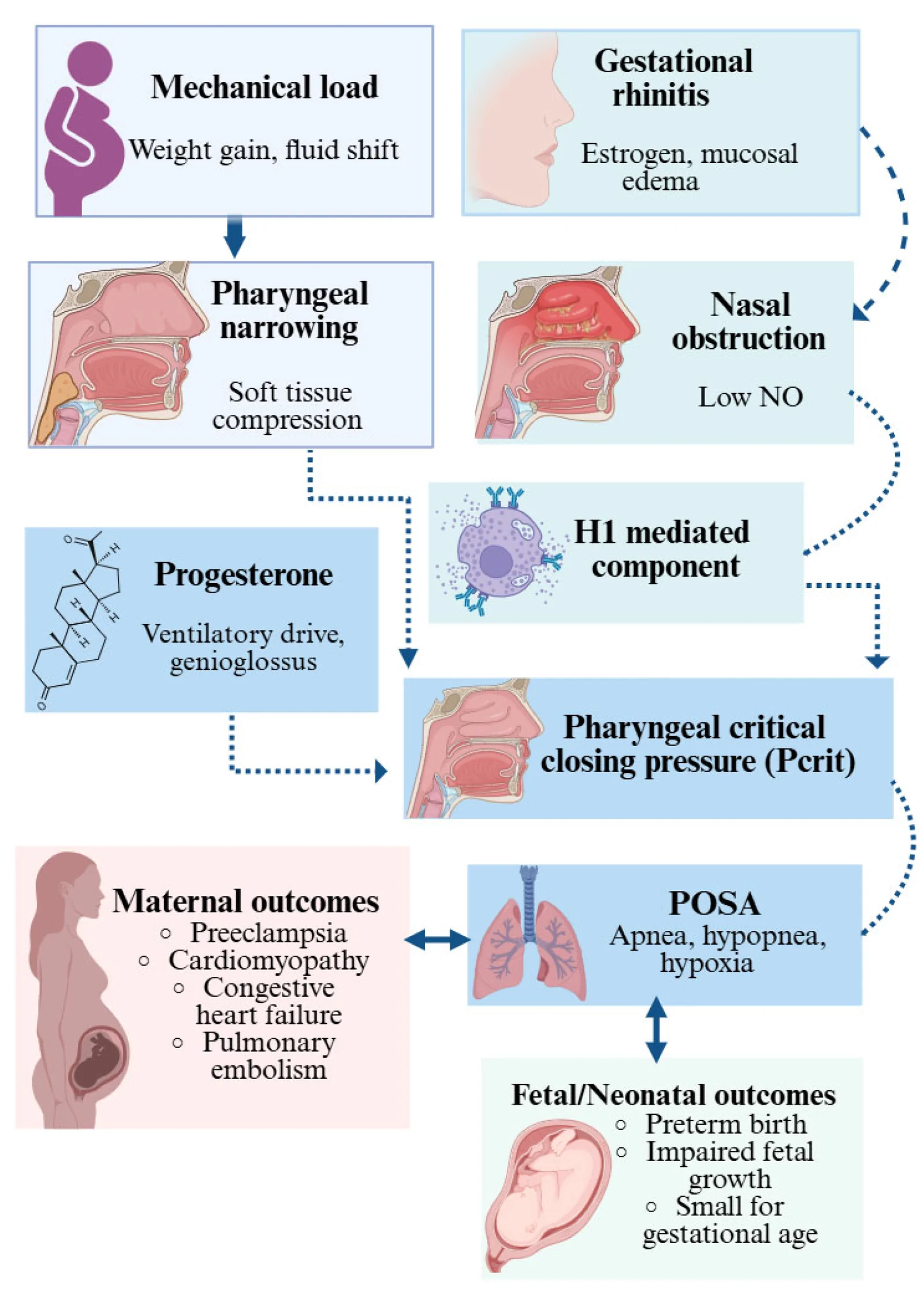
Hypothesis-generating conceptual model of pathways linking pregnancy-related changes to pregnancy-recognized OSA. Gestational weight gain, fluid redistribution, and hormonal changes are thought to increase pharyngeal collapsibility. Concurrently, estrogen-driven vascular engorgement is thought to cause nasal mucosal edema (gestational rhinitis), promoting oral breathing, reducing nasal nitric oxide delivery, and increasing upper-airway resistance; a superimposed H1-mediated mucosal component is hypothetical, and concurrent sneezing, rhinorrhea, or pruritus may instead reflect concomitant allergic rhinitis. Progesterone may augment genioglossus activity and ventilatory drive, providing a partial countervailing effect. We identified no prospective study testing the integrated rhinitis-to-POSA pathway in any cohort. Solid arrows represent pregnancy-specific evidence, dashed arrows represent information extrapolated from non-pregnant humans, and dotted arrows represent hypotheses without direct human evidence. Links from POSA to maternal and fetal outcomes represent observational associations, not established causal effects. H1  =  histamine H1; NO  =  nitric oxide; Pcrit  =  critical closing pressure; POSA  =  pregnancy-recognized obstructive sleep apnea—Figure 2 created with BioRender under license.

Histamine/H1 pathway. Gestational rhinitis is thought to be driven mainly by hormonally mediated submucosal vascular engorgement rather than IgE sensitization or mast-cell degranulation [9], a mechanism that H1 receptor antagonism would not be expected to address. We hypothesize that gestational estrogen may also increase mucosal histamine responsiveness. In nasal polyp tissue from non-pregnant subjects, estrogen and progesterone receptors were expressed by mast cells but not by other immune cells [26], a finding compatible with, but not demonstrating, hormonal modulation of mast-cell activity, and one that does not establish a role for H1-mediated mechanisms in the nasal obstruction of gestational rhinitis; a superimposed histamine-responsive mucosal component therefore remains hypothetical (Figure 2). The two levels are not contradictory: H1 antagonists cannot correct the vascular engorgement that defines gestational rhinitis, but in pregnant women who additionally present with sneezing, rhinorrhea, or nasal pruritus—symptoms that may reflect concomitant allergic rhinitis rather than a histaminergic mechanism specific to gestational rhinitis—H1 blockade may address that symptom domain. Any such role would be contributory rather than causally dominant, and we identified no clinical trial testing whether H1 antagonism measurably reduces nasal obstruction in gestational rhinitis. Section 6 addresses its clinical implications.

## 6. Pharmacological Management of the Nasal and Rhinitic Component

Nasal therapy during pregnancy encompasses three distinct therapeutic hypotheses that should not be blended: (1) that the improvement of nasal patency reduces pharyngeal collapsibility or AHI independently; (2) that it improves adherence to PAP therapy; and (3) that maternal–fetal outcomes improve. For OSA severity, the only nasal intervention evaluated, nasal dilator strips, did not improve SDB measures in pregnant women with a body mass index ≥ 27 kg/m^2^ and habitual snoring who were not selected for rhinitis [16]. According to the SASM-SOAP guideline, nasal congestion affects PAP adherence; saline and intranasal corticosteroids may be used to treat congestion. This recommendation is based on expert opinion [7]. We identified no studies regarding maternal or fetal outcomes. In this section, we consider gestational safety, physiological effects, and clinical efficacy separately.

### 6.1. Nasal Hygiene

Isotonic saline irrigation can improve nasal breathing [9] and mucociliary clearance and poses no known risks to the fetus. It is an appropriate first-line non-pharmacological measure and a suitable adjunct to any pharmacological regimen [24]. We identified no study of saline irrigation on OSA severity or maternal–fetal outcomes in pregnancy. According to expert opinion, saline irrigations may help nasal congestion during PAP therapy, although this has not been formally studied [7].

### 6.2. Intranasal Corticosteroids: Safety Evidence and Absence of OSA Efficacy

Safety evidence. Budesonide has the most extensive gestational safety dataset, derived primarily from inhaled budesonide exposure in asthma; evidence specific to intranasal formulations is more limited and should be interpreted as an extrapolation based on lower systemic absorption [27]. For fluticasone propionate and mometasone furoate, direct epidemiologic pregnancy data are limited or not well documented; however, intranasal corticosteroids are generally considered low-risk at therapeutic doses because of minimal systemic absorption [24]. The ACOG and ACAAI agree that inhaled corticosteroids can be safely used in pregnancy for moderate-to-severe rhinitis when nonpharmacologic measures are insufficient [22]. In a population-based cohort, first-trimester intranasal triamcinolone was not associated with overall congenital malformations (OR 0.88; 95% CI 0.60–1.28) but was associated with respiratory defects (OR 2.71; 95% CI 1.11–6.64; five exposed cases), a finding the authors considered potentially attributable to chance [28]. Overall, available safety data do not indicate an increased risk of major malformations at therapeutic doses, although agent-specific signals cannot be excluded; these data provide no evidence of benefit for airway or obstetric outcomes.

Efficacy evidence. The only randomized placebo-controlled trial identified in pregnancy rhinitis found that intranasal fluticasone propionate did not significantly improve nasal congestion compared with placebo (53 women treated for 8 weeks; no differences in symptom scores, nasal peak expiratory flow, or acoustic rhinometry) [29]. We identified no study examining whether intranasal corticosteroid use in pregnant women reduces AHI, pharyngeal collapsibility, or any maternal or fetal OSA-related endpoint. The role of intranasal corticosteroids in adherence to PAP therapy is suggested by expert opinion, but has not been studied in pregnancy [7]. Safety data for these agents must not be interpreted as evidence of efficacy.

Appropriate use context. Intranasal corticosteroids, particularly budesonide, may be considered for symptomatic nasal obstruction in pregnant adults on the basis of their gestational safety profile [24,27], although the only randomized trial in gestational rhinitis did not show efficacy [29]. Their intended role is nasal symptom management. It is unknown whether treating nasal obstruction affects upper-airway dynamics. They should not be presented as OSA therapy.

Topical decongestants. Oxymetazoline has minimal systemic absorption; however, in a case–control study including 12,734 infants with malformations and 7606 non-malformed controls, exploratory analyses identified a small number of associations with oral and intranasal decongestants, and the authors concluded that first-trimester use of specific oral and possibly intranasal decongestants may be associated with some infrequent birth defects [30]. Topical decongestants are considered only for short-term relief when safer alternatives are unavailable [31], which limits their relevance to the sustained nasal obstruction implicated in OSA. First-trimester use of specific oral decongestants has been associated with infrequent birth defects, including phenylephrine with endocardial cushion defects and phenylpropanolamine with ear defects and pyloric stenosis [30].

### 6.3. Chlorpheniramine: Adjunctive Option for H1-Mediated Rhinitic Symptoms, Safety Evidence, and Absence of OSA Efficacy Evidence

Chlorpheniramine, a first-generation H1 receptor antagonist, has the most extensive gestational safety record among oral antihistamines, supported by review-level evidence encompassing more than 1000 first-trimester exposures without a confirmed teratogenic signal [31]. A meta-analysis of 37 studies found no increased risk of major malformations with first-trimester H1 antihistamine exposure (cohort studies: OR 1.07, 95% CI 0.98–1.16; case–control studies: OR 1.05, 95% CI 0.90–1.23) [12]. Rhinitis practice parameters recognize chlorpheniramine as a pregnancy-compatible option with a well-documented safety record; it remains clinically available during pregnancy [13,32]. No study identified in our search evaluated chlorpheniramine for the nasal obstruction component of POSA, AHI reduction, or any maternal or fetal OSA-related endpoint.

Its use targets relief of histamine-mediated rhinitic symptoms (sneezing, rhinorrhea, or nasal pruritus), which in pregnancy may reflect concomitant allergic rhinitis, and does not constitute OSA management. We found no clinical trials on OSA severity, PAP adherence, or maternal–fetal outcomes.

## 7. Screening and Diagnosis

The 2023 SASM-SOAP guideline distinguishes four steps. Screening: universal OSA screening is not recommended in all pregnancies (Class III; Level C-EO). Identification of high-risk patients: it suggests screening in patients with BMI ≥ 30 (Class IIa; Level C-EO), and considering screening in those with hypertensive disorders or diabetes in the index or a prior pregnancy (Class IIb; Level C-EO), preferably in the first or second trimester. Questionnaire-based risk stratification: the Berlin questionnaire, STOP-BANG, Epworth Sleepiness Scale, and ASA checklist are not recommended as stand-alone screening tools (Class III; Level B-NR). Pregnancy-specific prediction models require further validation (Class IIb). Diagnostic testing and treatment: HSAT may be a reasonable diagnostic tool (Class IIb; Level B-NR), although portable monitoring is less sensitive than polysomnography in pregnancy. Treatment follows the standard of care for non-pregnant adults (Class IIb; Level C-EO) [7]. In a validation study of 30 obese women in late pregnancy, a type III home sleep test showed relatively high agreement with in-laboratory polysomnography (intraclass correlation coefficient 0.76 for automated scoring; categorical agreement 83.3%) [33].

In the SASM-SOAP meta-analysis, STOP-BANG had a pooled sensitivity of 0.58 and specificity of 0.82 for OSA in pregnancy [7]. A 2026 systematic review and meta-analysis of five cohort studies (1096 pregnant participants; 573 with objective sleep testing, predominantly home sleep apnea testing) reported a pooled sensitivity of 0.587 (95% CI 0.464–0.699) and a pooled specificity of 0.749 (95% CI 0.360–0.941) for a STOP-Bang score ≥ 3 at an AHI or respiratory event index ≥ 5 events/hour, with wide between-study variability in specificity; the authors of that review identified partial verification as the most frequent risk-of-bias concern among the included studies and concluded that STOP-Bang should not be used as a stand-alone screening tool in pregnancy [34]. Because the two syntheses differ in included studies, reference standards, and statistical models, their estimates are not directly comparable; both indicate that STOP-Bang alone would miss approximately four in ten affected pregnant women. A 2025 validation cohort study found that the modified STOP-Bang and Berlin questionnaires showed sensitivities of 0.77 and 0.64, respectively, and specificities of 0.64 and 0.64, respectively, for OSA detection in high-risk obstetric populations at approximately 28 weeks’ gestation, with negative predictive values of approximately 79% [35]. A sensitivity of 77% implies that questionnaire screening alone would miss about one in four affected women in this high-risk population; clinical judgment and objective testing remain essential complements to any screening instrument. The OSA case definition used in the validation study was an AHI ≥ 5 events per hour on objective sleep testing.

Reported OSA prevalence estimates during pregnancy vary widely across studies because of differences in diagnostic thresholds, gestational age at assessment, ascertainment methods, and population risk profiles [2,7]. Polysomnographic or HSAT-confirmed OSA, which measures physiological disease, and administratively coded OSA, which measures documentation and is biased toward more severe or symptomatic cases, are not directly comparable; therefore, cross-study pooling of these estimates is not appropriate [3].

## 8. Maternal and Fetal Outcomes

### 8.1. Maternal Outcomes

Because pre-pregnancy objective sleep data are rarely available, the evidence summarized in this section should be interpreted as representing OSA or SDB recognized during pregnancy rather than objectively confirmed de novo OSA. Associations of OSA with hypertensive disorders of pregnancy have been reported using different definitions: AHI-defined SDB on home sleep testing in a prospective cohort [20], and administratively coded OSA in national inpatient databases [1,3]. Because these approaches differ in sensitivity and in their ability to detect severe or symptomatic disease, we present their estimates separately rather than combined. A 2024 meta-analysis of four randomized and two observational studies (*n* = 809) by Lee et al. found CPAP use to be associated with a lower risk of gestational hypertension (RR, 0.65; 95% CI, 0.47–0.89; *p* = 0.008) and preeclampsia (RR 0.70, 95% CI 0.50–0.98; *p* = 0.04) [14]. In subgroup analyses, the reduction in gestational hypertension was statistically significant among randomized trials (RR, 0.57; 95% CI, 0.39–0.84) but not among observational studies, and the reduction in preeclampsia was not statistically significant in either subgroup; in leave-one-out analyses, the association with gestational hypertension lost statistical significance when the largest trial was omitted, and the association with preeclampsia weakened when any of three studies was omitted [14]. The randomized component also included a trial of a screening strategy in which few participants received positive airway pressure [34]. Both observational studies compared CPAP-adherent women with non-adherent or conservatively managed women, a comparison susceptible to healthy-adherer bias. These estimates should therefore be interpreted as associations rather than confirmed treatment effects; certainty remains limited by study size, variable adherence, heterogeneity, and potential residual confounding in the observational components. In the largest trial, an open-label multicenter study of high-risk pregnant women with mild-to-moderate OSA (340 randomized; 30 excluded after randomization; 310 analyzed), CPAP reduced diastolic blood pressure, the primary endpoint, by 2.2 mmHg and was associated with a lower incidence of preeclampsia, a secondary endpoint (13.1% vs. 22.3%; risk difference −9%; 95% CI, −18% to −1%), despite an adherence rate of 32.7% and mean use of 2.5 h/night; the trial was registered retrospectively [35]. Two other randomized trials of positive airway pressure found no statistically significant differences in their primary or main outcomes; both were small, adherence was low, and neither was designed to exclude clinically relevant effects. In 89 women with obesity and OSA randomized to autotitrating positive airway pressure or to sham or sleep-hygiene control, a composite of uterine artery Doppler pulsatility index, angiogenic ratio, and insulin resistance did not differ between groups, and adherence was low (mean use, 0.3–1.3 h/night) [36]. In a pilot trial in women with hypertensive disorders of pregnancy, 24-h blood pressure, arterial stiffness, and adverse maternal and fetal outcomes did not differ significantly between positive airway pressure and nasal dilator strips in either intention-to-treat or per-protocol analyses [37]. A separate trial evaluated a screening strategy rather than treatment efficacy: among 193 pregnant women at high risk of OSA randomized to sleep-study surveillance with targeted autotitrating positive airway pressure or to standard care without screening, composite adverse pregnancy outcomes did not differ significantly (46.4% vs. 43.3%; *p* = 0.77); because only six women were prescribed positive airway pressure and use ranged from 2% to 43% of days, this result informs the screening strategy but not the efficacy of adequately used positive airway pressure [34]. Adequately powered randomized controlled trials are required before the association reported in the meta-analysis can be attributed to a confirmed treatment effect.

National inpatient database analyses document higher rates of cardiomyopathy, congestive heart failure, pulmonary embolism, preterm delivery, and in-hospital mortality among women with administratively coded OSA [1,3]. These data reflect documentation at delivery rather than objective testing, and the associations are substantially confounded by pre-pregnancy obesity, gestational diabetes mellitus, and chronic hypertension. Adjusted analyses reduce but do not eliminate the observed associations, and causal inference is not supported [3].

Bourjeily et al. reported lower placenta-associated plasma protein A (PAPP-A) levels and a higher soluble fms-like tyrosine kinase 1 (sFlt-1) to placental growth factor (PlGF) ratio in 24 pregnant women with polysomnographically confirmed OSA (AHI > 5 events/hour) than in 166 controls at low risk for OSA, after adjustment for body mass index, findings consistent with antiangiogenic dysregulation as a candidate mediating pathway [21]. This case–control biomarker study does not establish causality.

During pregnancy, gestational weight gain is a potentially modifiable factor associated with SDB [2]. The NuMoM2b Sleep-Disordered Breathing sub-study found that AHI-defined SDB (AHI ≥ 5 events/hour level 3 home sleep testing, with hypopneas scored by ≥3% desaturation) in early and mid-pregnancy was independently associated with preeclampsia (adjusted OR 1.94; 95% CI 1.07–3.51 and 1.95; 95% CI 1.18–3.23, respectively) and gestational diabetes mellitus (adjusted OR 3.47; 95% CI 1.95–6.19 and 2.79; 95% CI 1.63–4.77), whereas the association with hypertensive disorders of pregnancy was statistically significant in mid-pregnancy (adjusted OR 1.73; 95% CI 1.19–2.52) but not in early pregnancy (adjusted OR 1.46; 95% CI 0.91–2.32), after adjustment for pre-pregnancy BMI, chronic hypertension, and other covariates [20]. Excessive gestational weight gain is a recognized contributor to increased OSA risk in pregnancy and may worsen upper-airway vulnerability through adverse respiratory mechanics [2]. Pre-pregnancy obesity is a major OSA risk factor and a predominant confounder in outcome studies; preconception weight optimization may be considered, although we identified no study of its effect on POSA in women at elevated metabolic risk [2,20].

Positional therapy, using devices that maintain lateral sleep position, reduced mean AHI by 11.3 events/hour in non-pregnant adults with positional OSA [15]. Pregnancy-specific efficacy remains unknown: we identified no trial in pregnant women. Its low risk and physiological plausibility do not establish clinical efficacy during pregnancy. It may be considered only as an adjunct to, rather than an alternative for, PAP therapy in patients with objectively documented positional worsening of OSA.

The cross-domain evidence synthesized above varies substantially in design quality, sample size, and confounding profile. Table 2 provides a descriptive, qualitative summary of design-related limitations by evidence domain. The ‘Certainty constraints’ column does not represent a formal GRADE rating risk-of-bias assessment.

Additionally, CPAP and other forms of positive airway pressure (PAP) therapy remain the primary treatment for moderate-to-severe OSA in pregnancy [7]. Evidence on maternal hypertensive outcomes is summarized earlier in this section. Mean nightly CPAP use in published pregnancy trials has ranged from less than 1 to approximately 3 h [17,37,39], below the conventional threshold of 4 h per night used to define adherence [37]. Per-protocol and dose–response analyses in some trials suggest larger effects with greater use [16,39], whereas another trial found no statistically significant differences in either intention-to-treat or per-protocol analyses [37]; intention-to-treat estimates may therefore underestimate the benefit achievable with adequate adherence; however, per-protocol estimates may be biased by adherence-related selection, as adherent participants may differ from non-adherent participants in disease severity, comorbidity profile, or motivation, and should be interpreted alongside intention-to-treat analyses.

### 8.2. Fetal and Neonatal Outcomes

Evidence reported by Fung et al. suggests an association between maternal OSA during pregnancy and impaired fetal growth; they screened 371 women in the second trimester and performed near-term polysomnography in 41, stratified by questionnaire-based OSA risk [4]. Impaired fetal growth—defined as a composite of birthweight below the 10th customized centile or a fall in customized centile exceeding one-third between 32 weeks and delivery—occurred in 6 of 14 women with confirmed OSA (defined as a respiratory disturbance index ≥ 5 events/hour); all sleep studies were performed at approximately 37 weeks’ gestation to minimize the risk of iatrogenic preterm delivery triggered by abnormal fetal heart rate monitoring at a preterm gestation. Compared with 3 of 27 without OSA, yet confounding remains important. On univariate logistic regression, OSA was the strongest predictor of the composite outcome (OR 6.00, 95% CI 1.2–29.7; *p* = 0.03); after adjustment for pre-pregnancy BMI, the point estimate was attenuated and did not retain conventional statistical significance (adjusted OR 5.30, 95% CI 0.93–30.34; *p* = 0.061), indicating that independent confirmation in larger cohorts is required before the OSA-fetal growth association can be regarded as established [4]. The difference between groups was driven primarily by slowing of third-trimester growth trajectories (4/14 OSA vs. 0/27 controls) rather than by birthweight below the 10th centile alone (2/14 vs. 3/27). In that study, researchers observed prolonged fetal heart rate decelerations during maternal oxygen desaturation in a single fetus later identified with severe growth restriction; acute fetal heart rate responses did not accompany most respiratory events, and causality remains unestablished [4].

The largest prospective cohort study of SDB in pregnancy that we identified enrolled 3705 nulliparous women and conducted blinded in-home sleep testing at 6–15 weeks’ and 22–31 weeks’ gestation, defining SDB as an AHI ≥ 5 events/h with hypopneas scored by ≥3% desaturation; valid AHI data were available for 3132 participants (84.5%) in early pregnancy and 2474 (66.8%) in mid-pregnancy [20]. The figure of 2106 participants in the Introduction refers to the analytic sample of that cohort used in the neonatal composite outcome analysis [5]. In the 2106-participant analytic subsample, early-pregnancy AHI-defined SDB showed an unadjusted association with the neonatal composite outcome that was no longer statistically significant after full covariate adjustment (maternal age, chronic hypertension, pregestational diabetes, and BMI), whereas new-onset mid-pregnancy AHI-defined SDB retained an independent adjusted association (adjusted RR 1.43, 95% CI 1.05–1.94); residual confounding by other obesity-related metabolic comorbidities cannot be excluded [5].

Blyton et al. found markedly fewer overnight fetal movements in 20 women with preeclampsia than in 20 normotensive controls (319 ± 32 vs. 689 ± 160 movements; *p* < 0.001); in 10 additional women with preeclampsia studied on a control night and on a night of nasal CPAP, without randomization, CPAP was associated with higher fetal movement counts (*p* < 0.001) and fetal hiccups (*p* = 0.0002) [18]. In an earlier parallel-group RCT of 24 women with severe preeclampsia, nocturnal cardiac output was higher in the CPAP group, and nocturnal cardiac output correlated with neonatal birthweight (R^2^ = 0.64; equivalent to r ≈ 0.80; *p* < 0.001) [17]. These observations are consistent with a possible treatment-related improvement in fetal physiology. Still, very small sample sizes, a preeclampsia-specific population, and the absence of a validated sham condition across all arms limit these findings and do not support generalizable conclusions. In these studies, the exposure was preeclampsia with SDB, not objectively confirmed OSA; these findings cannot be extrapolated to women with OSA without preeclampsia.

Chirakalwasan et al. randomized 36 obese pregnant women (24–34 weeks’ gestation) with diet-controlled gestational diabetes mellitus and OSA (respiratory event index ≥ 5 events/hour on a portable monitor) to two weeks of nightly CPAP or waitlist control [16]. The primary glucose metabolism endpoint showed no significant between-group differences. In post-hoc analyses, women who used CPAP for longer than 2 weeks had lower rates of preterm delivery (*p* = 0.002), unplanned cesarean section (*p* = 0.005), and neonatal intensive care unit (NICU) admission (*p* < 0.001) than those who did not [16]; this comparison was defined after randomization, and mean adherence during the trial was 46.7% of prescribed nightly use (4.45 h on the days of use and 3.39 h across all days). Because these analyses were not pre-specified and adherence was suboptimal, the findings should be interpreted as exploratory rather than confirmatory. The absence of multiplicity adjustment and the restriction of per-protocol analyses to adherent participants may limit these comparisons. The largest published RCT identified (340 randomized; 310 analyzed) compared CPAP with usual care in high-risk pregnant women with OSA, with blood pressure as the primary endpoint and preeclampsia as a secondary endpoint [39]; Table 3 summarizes the randomized trials identified. Across the trials we identified, neonatal endpoints were underpowered [14,16,17,18,34,36,37,39]; the ongoing multicenter trial (NCT03487185; target *n* = 1500), sponsored by The George Washington University Biostatistics Center, has hypertensive disorders of pregnancy as its primary endpoint, with neonatal outcomes as pre-specified secondary endpoints, and is expected to provide more definitive data on both maternal and neonatal outcomes [40].

In a single-center retrospective case–control analysis of 31 women with untreated, polysomnographically confirmed OSA (AHI ≥ 5 events/hour) and 48 women without OSA, Kneitel et al. found no difference in the proportion of infants with birthweight below the 10th customized centile (23% vs. 25%); however, OSA was associated with impaired fetal growth (adjusted OR 3.9; 95% CI 1.2–12.6), driven mainly by slowing of fetal growth across the third trimester (61% vs. 29%) [38]. The retrospective design and small sample size cannot exclude residual confounding by obesity-related comorbidities.

## 9. Discussion

This review integrates separate literature into a hypothesis-generating conceptual model of POSA.

### 9.1. Observational Associations and Guideline Recommendations

OSA or SDB recognized during pregnancy is associated with hypertensive disorders of pregnancy and severe maternal morbidity across observational studies using heterogeneous definitions [1,3,20]. On the basis of limited data and expert opinion, current guidance recommends CPAP for objectively confirmed OSA to reduce AHI and OSA symptoms (Class IIa; Level C-LD/EO) [7].

### 9.2. Suggestive but Uncertain Evidence

It is unknown whether OSA treatment improves pregnancy outcomes. A mixed-design meta-analysis found CPAP use to be associated with a lower risk of gestational hypertension and preeclampsia, although the preeclampsia estimate was not robust in subgroup and sensitivity analyses [14]; one open-label randomized trial reported reductions in diastolic blood pressure and preeclampsia incidence [39], whereas other small trials of positive airway pressure found no statistically significant differences in cardiometabolic, glycemic, or blood pressure outcomes [16,36,37], and a trial of a screening strategy with targeted treatment did not reduce composite adverse pregnancy outcomes [34]. Evidence remains limited by small sample sizes, low adherence, and the inclusion of observational designs, and no trial has been adequately powered for neonatal outcomes. Non-significant results from these trials therefore do not demonstrate absence of benefit.

### 9.3. Evidence Extrapolated from Non-Pregnant Population

Evidence focuses on the effects of oral breathing on pharyngeal collapsibility, the effects of progesterone on genioglossus activity, positional therapy on AHI, and NO production in the sinuses derived from non-pregnant adults [6,15,25]. These data provide a physiological basis, but not pregnancy-specific evidence.

### 9.4. Newly Hypothesized

The pathway in Figure 2 is proposed as a testable hypothesis linking gestational rhinitis to pregnancy-recognized OSA through nasal obstruction and pharyngeal collapsibility, with a hypothetical superimposed H1-mediated mucosal component. This has not been directly evaluated; we identified no study testing it in pregnant adults. Pregnancy-compatible rhinitis therapies may be used to reduce nasal symptoms when nasal congestion limits CPAP tolerance and adherence, rather than as a specific OSA therapy, as suggested by expert opinion [7]. Effects on PAP adherence, OSA severity, and maternal–fetal outcomes remain untested.

## 10. Research Gaps and Future Directions

This synthesis identifies three primary domains as priority areas for prospective investigation.

Gestational rhinitis, nasal physiology, and upper-airway collapsibility in pregnant women with SDB. Prospective gestational cohort studies integrating objective OSA phenotyping with nasal physiology, upper-airway collapsibility, and nasal nitric oxide measurement are needed to test the proposed rhinitis-to-OSA framework. Whether oral breathing secondary to gestational nasal obstruction alters the oral microbiome during pregnancy has not been studied; this question merits investigation because maternal oral dysbiosis and periodontal pathogens have been associated with preterm birth and low birthweight in observational studies, although methodological heterogeneity limits comparability [19].

Across the studies we identified, trials of CPAP or positive airway pressure therapy in pregnant women with OSA had secondary, exploratory, or unspecified neonatal endpoints, and no individual trial was powered to detect clinically meaningful differences in SGA rate, preterm birth, or NICU admission. The ongoing multicenter trial (NCT03487185; target *n* = 1500), sponsored by The George Washington University Biostatistics Center, has hypertensive disorders of pregnancy as its primary endpoint and includes neonatal outcomes as pre-specified secondary endpoints; results are not yet available [40]. Future trial design should pre-specify neonatal outcomes (SGA + preterm birth < 37 weeks + NICU admission > 48 h) as a co-primary endpoint alongside maternal hypertensive outcomes, include objective CPAP adherence monitoring with per-protocol and intention-to-treat analyses reported separately, stratify by pre-pregnancy BMI category, and incorporate third-trimester uterine artery Doppler measurements as a mechanistic secondary endpoint.

Screening implementation and clinical translation. Screening questionnaires developed for non-pregnant populations, such as STOP-Bang and the Berlin questionnaire, show limited accuracy in pregnancy (pooled STOP-Bang sensitivity of approximately 0.58 in two meta-analyses [7,41]; sensitivities of 0.64–0.77 in a high-risk obstetric cohort [35]) and are not recommended as stand-alone screening tools [7], leaving a substantial proportion of affected women undetected. A stepped-wedge cluster-randomized implementation trial embedding HSAT-based OSA screening into routine prenatal care visits at 24–28 weeks’ gestation—with downstream referral pathways and treatment linkage as the experimental condition—would measure the effect of systematic identification on diagnosis rates, treatment uptake, and maternal–fetal outcomes at the health-system level.

## 11. Limitations

Several limitations of this review required explicit acknowledgment; each informed how evidence was weighted in the synthesis.

First, we conducted the structured search in PubMed. We did not search the Cochrane Library, Embase, or other sources. We mitigated this by relying, where possible, on systematic reviews, meta-analyses, and guidelines that searched additional databases and by hand-searching reference lists. Reference lists of included articles were hand-searched to identify additional sources. Targeted verification during revision identified six sources not retrieved by the original strategy [10,29,34,36,41,42], indicating incomplete search sensitivity. Statements that no study addressed a question therefore refer only to studies identified by our search. The English-language restriction may also have introduced language bias. As with all narrative reviews, we cannot exclude publication bias.

Second, reported OSA prevalence estimates during pregnancy vary widely, reflecting heterogeneity in diagnostic criteria, AHI thresholds, gestational age at assessment, ascertainment methods, and population risk profiles [2,7]. Polysomnographic or HSAT-confirmed OSA and administratively coded OSA measure distinct constructs and are not directly comparable; cross-study pooling of these estimates is inappropriate. Third, the relationship between OSA and adverse pregnancy outcomes is substantially confounded by obesity and associated metabolic comorbidities. Pre-pregnancy obesity, gestational diabetes mellitus, and chronic hypertension independently elevate both OSA risk and maternal–fetal morbidity. However, residual confounding is likely even after adjustment, given the high collinearity between OSA and obesity.

Fourth, CPAP adherence in pregnancy is suboptimal in most published trials, and therapeutic thresholds derived from non-pregnant populations may not translate directly to the obstetric context. Several trials incompletely reported adherence data, limiting interpretation of treatment effect estimates as reflecting true per-protocol efficacy.

Fifth, the gestational rhinitis–SDB chain, including the proposed role of H1-mediated pathways, is informed by individual mechanistic observations from tissue and non-pregnant physiological studies, but has not been tested as an integrated pathway in any pregnant cohort. We identified no trial of rhinitis therapy with OSA-related endpoints. As noted above, the search was limited to PubMed; these statements refer to studies identified in our search. The mechanistic sections motivate future research design, not clinical recommendations.

Finally, narrative synthesis is inherently selective; we used a domain-organized search structure and dual-reviewer screening to reduce selection bias. All mechanistic claims are explicitly framed as hypothesis-generating throughout the text, and the evidence supporting each domain is transparently qualified by study design and confounding profile. In addition, because this was a qualitative appraisal, the evidence weighting reflects the authors’ judgment and was not based on a formal risk-of-bias or certainty assessment.

## 12. Conclusions

OSA and SDB recognized during pregnancy are associated in observational studies with hypertensive disorders, preterm birth, and increased maternal morbidity, and possibly with impaired fetal growth; however, pregnancy-specific studies are small, definitions are heterogeneous, and residual confounding by obesity and metabolic comorbidities precludes causal inference. PAP therapy is the principal treatment for objectively confirmed OSA, although randomized evidence that it improves pregnancy outcomes is limited and inconsistent. Gestational rhinitis therapies should be considered as nasal symptom management rather than OSA treatment. The proposed conceptual model is hypothesis-generating, and its links should be tested in prospective cohorts of pregnant women and in randomized controlled trials with prespecified maternal and neonatal outcomes.

## Figures and Tables

**Table 1:** Key terminology: distinguishing related but non-equivalent constructs used in this review. Prevalence estimates derived from different ascertainment methods reflect distinct constructs and should be interpreted within their respective study contexts.

Construct	Definition	Ascertainment	Interpretive Caution
SDB	Full clinical spectrum from habitual snoring to OSA.	Questionnaires, Polysomnography (PSG), HSAT.	A prevalence of 17–45% in the third trimester reflects the SDB spectrum and should not be equated with OSA prevalence [2].
PSG/HSAT-confirmed OSA	AHI ≥ 5/h on objective sleep testing + symptoms or comorbidity.	PSG or HSAT.	Measures physiological disease; not comparable with administrative coding rates [2,3].
Administratively coded OSA	International Classification of Diseases-coded OSA at hospital discharge.	Claims/inpatient databases.	Measures documentation frequency, not prevalence; biased toward severe/symptomatic cases [1,3].
Pregnancy-recognized OSA (diagnostic construct)	OSA is first recognized during pregnancy, regardless of the true onset.	Incident diagnosis during gestation.	Diagnostic circumstance, not a distinct biological phenotype; pre-pregnancy PSG data rarely available [1].

AHI = apnea-hypopnea index; HSAT = home sleep apnea testing; OSA = obstructive sleep apnea; PSG = polysomnography; SDB = sleep-disordered breathing.

**Table 2:** Evidence domains, principal study designs, populations, and key limitations for claims advanced in this review. Observational evidence on OSA and pregnancy outcomes is presented separately from interventional evidence on positive airway pressure therapy. AHI = apnea-hypopnea index; BMI = body mass index; CPAP = continuous positive airway pressure; GDM = gestational diabetes mellitus; HSAT = home sleep apnea testing; MA = meta-analysis; NO = nitric oxide; OR = odds ratio; OSA = obstructive sleep apnea; PAP = positive airway pressure; PSG = polysomnography; RCT = randomized controlled trial; RDI = respiratory disturbance index; SDB = sleep-disordered breathing; SR = systematic review. Safety data for pharmacological agents are evaluated separately from efficacy data; the lack of a teratogenic signal does not imply demonstrated therapeutic benefit for any OSA-related endpoint.

Evidence Source/Type	Population	Exposure Definition	Directness to Pregnancy	Main Limitations	Certainty Constraints
Observational evidence on OSA and maternal outcomes: prospective cohort with home sleep testing [20]; administrative database studies [1,3]; case–control biomarker study [21]	Pregnant women; nulliparous cohort; hospital deliveries	AHI-defined SDB [20]; PSG-confirmed OSA [21]; administratively coded OSA [3]	Direct	Substantial confounding by obesity, GDM, and chronic hypertension; administrative data measure documentation, not disease; heterogeneous OSA definitions	Associations only; causal inference not supported
Observational evidence on OSA and fetal or neonatal outcomes: small prospective PSG cohort (*n* = 41 with sleep study) [4]; retrospective case–control study [38]; NuMoM2b SDB sub-study (HSAT; 2106 in the neonatal analysis) [5]	Pregnant women; small high-risk samples; nulliparous cohort	RDI-defined OSA on PSG [4]; AHI-defined OSA on PSG [38]; AHI-defined SDB on HSAT [5]	Direct	Small samples; BMI-adjusted OR for fetal growth non-significant (OR 5.30; *p* = 0.061) [4]; residual confounding by obesity-related comorbidities	Possible association with impaired fetal growth; independent confirmation required
Interventional evidence on positive airway pressure therapy: SR/MA of mixed RCT and observational studies [14]; RCTs [16,17,36,37,39]; RCT of a screening strategy with targeted treatment [34]; non-randomized crossover study [18]	Pregnant women with OSA or SDB, mostly high-risk or obese; preeclampsia [17,18]	CPAP or autotitrating PAP vs. usual care, waitlist, sham, sleep hygiene, or nasal dilator strips	Direct	Small trials; low adherence; open-label designs and post-randomization exclusions; adherence-based comparisons in observational components and post hoc analyses; neonatal endpoints underpowered	Preliminary association with hypertensive outcomes, not robust in subgroup and sensitivity analyses; no trial powered for neonatal outcomes
Pharmacological and physiological studies in non-pregnant adults; computational fluid dynamics modeling	Non-pregnant adults; one small preeclampsia subgroup (not rhinitis-specific)	Not applicable	Non-pregnant human	We identified no study characterizing nasal exhaled NO in pregnant women with gestational rhinitis or SDB; the physiological role of entrained nasal NO during tidal breathing remains debated.	Unconfirmed in any gestational rhinitis or pregnancy-recognized OSA cohort

**Table 3:** Randomized trials of positive airway pressure therapy in pregnancy, and one randomized trial of a screening strategy with targeted treatment, identified for this review. APAP = autotitrating positive airway pressure; AHI = apnea-hypopnea index; BMI = body mass index; BP = blood pressure; CPAP = continuous positive airway pressure; DBP = diastolic blood pressure; GDM = gestational diabetes mellitus; HDP = hypertensive disorders of pregnancy; HOMA-IR = homeostatic model assessment of insulin resistance; ITT = intention-to-treat; NICU = neonatal intensive care unit; OSA = obstructive sleep apnea; PAP = positive airway pressure; RCT = randomized controlled trial; RDI = respiratory disturbance index; sFlt-1/PlGF = soluble fms-like tyrosine kinase-1 to placental growth factor ratio. The non-randomized crossover study of CPAP in preeclampsia [18] is described in the text.

Trial (Design)	Population (n Randomized)	Intervention vs. Comparator	Primary Outcome or Main Measure	Adherence	Main Findings
Blyton et al., 2004 [17] (RCT)	Women with severe preeclampsia (*n* = 24)	Nocturnal nasal CPAP vs. no treatment	Nocturnal hemodynamics (cardiac output) during polysomnography	Not reported	Sleep-related reductions in cardiac output were minimized with CPAP; nocturnal cardiac output correlated with birthweight (r^2^ = 0.64)
Chirakalwasan et al., 2018 [16] (RCT)	Obese women with diet-controlled GDM and OSA at 24–34 weeks (*n* = 36)	CPAP for 2 weeks vs. waitlist control	Glucose metabolism (meal tolerance test)	46.7% of prescribed nightly use; <5 events/hour	No statistically significant between-group difference in glucose metabolism; lower preterm delivery, unplanned cesarean, and NICU admission among women who continued CPAP > 2 weeks (post hoc, non-randomized comparison)
Kalkhoff et al., 2022 [34] (RCT of a screening strategy)	Women at high risk of OSA (*n* = 193)	Sleep-study surveillance with targeted APAP vs. standard care without screening	Composite adverse pregnancy outcomes	APAP prescribed to 6 women; use on 2–43% of days	No statistically significant difference (46.4% vs. 43.3%; *p* = 0.77); evaluates the screening strategy, not treatment efficacy
Facco et al., 2023 [36] (RCT)	Women with BMI ≥ 30 kg/m^2^ and OSA (AHI 5–50) at 14–20 weeks (*n* = 89)	APAP vs. sham CPAP (phase 1) or sleep hygiene (phase 2)	Composite of uterine artery Doppler pulsatility index, sFlt-1/PlGF ratio, and HOMA-IR	Low (mean 0.3–1.3 h/night)	No statistically significant difference in the primary outcome; lower uterine artery pulsatility index vs. sleep hygiene in a secondary analysis
Tantrakul et al., 2023 [39] (open-label RCT; retrospectively registered)	High-risk pregnant women with mild-to-moderate OSA (RDI 5–29.99), mostly recruited in the first trimester (*n* = 340; 310 analyzed)	CPAP vs. usual care	Blood pressure	32.7%; mean 2.5 h/night	DBP −2.2 mmHg (95% CI −3.9 to −0.4); preeclampsia 13.1% vs. 22.3% (secondary endpoint)
Panyarath et al., 2024 [37] (pilot RCT)	Women with HDP and mild-to-moderate OSA (*n* = 48)	PAP vs. nasal dilator strips	Feasibility (recruitment and adherence)	Mean 3.1 h/night; ≥4 h on 38% of nights	No statistically significant differences in 24-h BP, arterial stiffness, or maternal and fetal outcomes (ITT and per-protocol)

## Data Availability

No new data were created or analyzed in this study. Data sharing is not applicable to this article.

## Appendix A

### Appendix A.1

Complete PubMed search strategy by domain. PubMed was searched for this structured narrative review. Six clinical and mechanistic domains organized the search strategy to ensure coverage of the sleep medicine, obstetrics, rhinology, and pharmacovigilance studies central to this review. Potential under-representation of literature indexed predominantly in Embase and the Cochrane Library is noted in Section 11. Searches were conducted on 7 March 2026. On 9 October 2026, targeted supplementary searches (not a full repetition of these strings) were performed as described in Section 2. MeSH = Medical Subject Headings; tiab = title and abstract field tag; AND/OR = Boolean operators; [MeSH] = controlled vocabulary term. All searches included an English-language filter. Human-study filter applied to clinical outcome domains; no species filter applied to mechanistic domains (iii, iv) to capture animal-model evidence. This review was conducted in PubMed.

**Table A1:** Complete PubMed search strategy.

Domain	Search String (PubMed Syntax)	Filters Applied	Rationale for Inclusion
OSA/SDB in pregnancy (core)	(“obstructive sleep apnea”[MeSH] OR “sleep apnea, obstructive”[MeSH] OR “sleep-disordered breathing”[tiab] OR “obstructive sleep apnea”[tiab] OR “sleep apnea”[tiab]) AND (“pregnancy”[MeSH] OR “pregnant women”[MeSH] OR “pregnancy complications”[MeSH] OR “gestational”[tiab] OR “prenatal”[tiab] OR “obstetric”[tiab])	English; humans; inception–7 March 2026	Primary clinical domain; no year restriction for primary studies
Gestational rhinitis	(“rhinitis”[MeSH] OR “nasal obstruction”[MeSH] OR “gestational rhinitis”[tiab] OR “pregnancy rhinitis”[tiab]) AND (“pregnancy”[MeSH] OR “pregnant”[tiab])	English; inception–7 March 2026	Upper-airway collapsibility mechanism in pregnancy; H1 pathway plausibility
Nitric oxide and airway/vascular function	(“nitric oxide”[MeSH] OR “nitric oxide”[tiab]) AND (“airway”[tiab] OR “blood pressure”[MeSH] OR “endothelial”[tiab] OR “oral bacteria”[tiab] OR “nitrate-reducing”[tiab])	English; no year restriction; humans and animal models	Foundational mechanistic data for the NO pathway; no gestational-specific literature exists for most sub-questions
Nasal pharmacotherapy in pregnancy	(“intranasal corticosteroids”[tiab] OR “nasal decongestants”[tiab] OR “oxymetazoline”[tiab] OR “budesonide”[tiab] OR “antihistamines”[tiab] OR “chlorpheniramine”[tiab]) AND (“pregnancy”[MeSH] OR “pregnant”[tiab])	English; inception–7 March 2026	Drug safety domain: direct evidence in pregnant populations prioritized where available.
CPAP and PAP therapy in pregnancy	(“continuous positive airway pressure”[MeSH] OR “CPAP”[tiab] OR “positive airway pressure”[tiab]) AND (“pregnancy”[MeSH] OR “pregnant”[tiab]) AND (“blood pressure”[MeSH] OR “preeclampsia”[MeSH] OR “hypertension”[MeSH] OR “outcome”[tiab])	English; no year restriction; RCTs and systematic reviews prioritized	Intervention domain: direct evidence on the use of CPAP and PAP therapy.
Screening and diagnosis (HSAT, questionnaires)	(“home sleep testing”[tiab] OR “HSAT”[tiab] OR “polysomnography”[MeSH] OR “STOP-Bang”[tiab] OR “Berlin questionnaire”[tiab]) AND (“pregnancy”[MeSH] OR “pregnant”[tiab] OR “obstetric”[tiab])	English; inception–7 March 2026	Diagnostic validity domain: primary studies in pregnant populations

### Appendix A.2

Literature selection flow. Two independent reviewers (C.A.P. and K.G.Z.) performed all screening steps. Disagreements were resolved by discussion. This table describes the selection flow from the PubMed search to the structured evidence set and the final reference list. The final manuscript cites 42 references: the structured evidence set, six sources identified during revision [10,29,34,36,41,42], and three references added outside the structured PubMed search (one clinical review [9], one methodological reference [11], and one systematic review suggested during peer review [19]).

**Table A2:** Literature selection flow.

Step	Records (Identified/Removed)	Cumulative *n*	Notes
PubMed search (MeSH + keyword combinations)	84 records identified	84	Search date: 7 March 2026
Duplicate removal	13 duplicates removed	71	Manual deduplication by C.A.P.
Title and abstract screening (C.A.P., K.G.Z.)	33 excluded (off-topic, non-English, editorials without data)	38	Disagreements resolved by discussion
Full-text review	5 excluded (insufficient methods; data not extractable)	33	Final included structured evidence set: 33 studies and guidelines
Additional records identified during revision (targeted verification, 9 October 2026)	6 records [10,29,34,36,41,42]	39	Identified through targeted verification of statements of absence of evidence of prevalence and diagnostic accuracy estimates, and of the trials included in a published meta-analysis [14].
References added outside the structured search	3	42	One clinical review [9], one methodological reference [11], and one systematic review suggested during peer review [19]
Final references cited	-	42	33 + 6 + 3 = 42

### Appendix A.3

SANRA item mapping. SANRA = Scale for the Assessment of Narrative Review Articles. Items 1–6 correspond to the SANRA scoring instrument [11], which rates each item from 0 (low standard) to 2 (high standard) and is intended for quality assessment by editors and reviewers. The table indicates where each item is addressed in the manuscript and does not constitute an independent quality rating.

**Table A3:** SANRA item mapping.

SANRA Item	Item Description	Location in the Manuscript	Addressed in Manuscript
1	Justification of the article’s importance for the readership	Introduction; the review addresses a clinically underrecognized condition with documented maternal–fetal consequences	Yes
2	Statement of concrete aims or formulation of questions	Introduction (final paragraph) and Methods; four integrated aims stated	Yes
3	Description of the literature search	Methods; domain-organized PubMed search with full Boolean strings (Appendix A.1) and selection flow (Appendix A.2)	Yes
4	Referencing	Key statements are referenced throughout the text, including methodological and trial-registry sources	Yes
5	Scientific reasoning, including presentation of the level of evidence	Mechanistic claims are framed as hypothesis-generating; treatment estimates are qualified by design, adherence, and confounding; Table 2 summarizes design, exposure definition, directness, limitations, and certainty constraints by domain	Yes
6	Appropriate presentation of relevant endpoint data	Effect estimates with confidence intervals and sample sizes are reported for key studies in Section 8 and Section 9 and in Table 3	Yes
